# HCV-Mediated Apoptosis of Hepatocytes in Culture and Viral Pathogenesis

**DOI:** 10.1371/journal.pone.0155708

**Published:** 2016-06-09

**Authors:** Erica Silberstein, Laura Ulitzky, Livia Alves Lima, Nicoleta Cehan, Andréa Teixeira-Carvalho, Philippe Roingeard, Deborah R. Taylor

**Affiliations:** 1 Laboratory of Emerging Pathogens, Division of Emerging Transfusion Transmitted Diseases, Office of Blood Research and Review, CBER FDA, Silver Spring, MD, 20903, United States of America; 2 INSERM U966, Universite Francois Rabelais and CHRU de Tours, Tours, France; SAINT LOUIS UNIVERSITY, UNITED STATES

## Abstract

Chronic Hepatitis C Virus (HCV) infection is associated with progressive liver injury and subsequent development of fibrosis and cirrhosis. The death of hepatocytes results in the release of cytokines that induce inflammatory and fibrotic responses. The mechanism of liver damage is still under investigation but both apoptosis and immune-mediated processes may play roles. By observing the changes in gene expression patterns in HCV-infected cells, both markers and the causes of HCV-associated liver injury may be elucidated. HCV genotype 1b virus from persistently infected VeroE6 cells induced a strong cytopathic effect when used to infect Huh7.5 hepatoma cells. To determine if this cytopathic effect was a result of apoptosis, ultrastructural changes were observed by electron microscopy and markers of programmed cell death were surveyed. Screening of a human PCR array demonstrated a gene expression profile that contained upregulated markers of apoptosis, including tumor necrosis factor, caspases and caspase activators, Fas, Bcl2-interacting killer (BIK) and tumor suppressor protein, p53, as a result of HCV genotype 1b infection. The genes identified in this study should provide new insights into understanding viral pathogenesis in liver cells and may possibly help to identify novel antiviral and antifibrotic targets.

## Introduction

Hepatitis C Virus (HCV) is a small enveloped virus belonging to the family Flaviviridae, genus Hepacivirus. The single positive-sense RNA genome encodes a polyprotein that is cleaved by viral and cellular proteases into 10 different proteins [1–4]. Due to its high genetic variability, HCV has been classified in six genotypes that are differentiated based on nucleotide sequence diversity [4–6]. Genotype 1a and 1b are associated with more chronic disease than any of the other genotypes [7].

Chronic HCV infection is associated with inflammatory liver damage and long term viral persistence resulting in a high risk of developing steatosis, fibrosis, cirrhosis, and hepatocellular carcinoma [8]. Abnormal retention of lipids results in adipose degeneration, or steatosis, a common feature of HCV infection. Cirrhosis is the irreversible endpoint of fibrosis, which is characterized by extensive scar formation, and an increase in the distribution of extracellular matrix components.

It is estimated that 2–3% of the worldwide population is persistently infected with HCV [4;9]. Most infections are asymptomatic, associated with only non-specific and mild symptoms, and therefore patients are diagnosed only after liver disease has already developed. The standard of care includes the use of pegylated interferon with ribavirin, but this is ineffective for about 50% of patients infected with genotype 1, the most common in the U.S., Europe and Japan [4;10]. The addition of a protease and replicase inhibitors have become the standard of care and have improved treatment outcomes greatly [11].

HCV-mediated liver injury is presumably caused by a diverse and complex array of factors. These factors include viral gene products that have direct intracellular and extracellular effects on apoptosis, steatosis and immune-mediated processes. The ultimate outcome of HCV infection depends on a complex balance of multiple competing factors. HCV proteins can cause steatosis [12], activate stellate cells leading to fibrosis [13], inhibit the intracellular interferon response to infection [14;15], and modulate apoptosis leading to hepatocellular carcinoma [16;17]. These effects are overlapping and interrelated [4]. Currently, liver biopsy is the most common method of assessing liver injury, but it is invasive, can be painful and is associated with a risk for serious complications. Research focused on non-invasive methods for the evaluation of liver fibrosis is needed in order to prevent the progression to cirrhosis.

Gene profiling analysis of HCV-infected cells can provide insight into the host factors that are essential for viral replication, involved in antiviral responses, and contribute to liver pathologies. Microarray expression profiling has been used to study host-gene expression in cells transfected with RNA encoding individual HCV genes, HCV subgenomic or full-length replicons, and in cells infected with HCV J6/JFH-1 virus [16;18–24]. These studies demonstrated that replication of HCV results in the regulation of a number of host genes involved in oxidative stress, apoptosis, lipid metabolism, immunity, proliferation, and intracellular transport [24]. A recent study investigated and compared the gene expression profiles in liver biopsy tissue from patients with fibrosis and cirrhosis resulting from HCV genotype 3a infection [18]. The authors noted significant changes in the expression of genes involved in cell signalling, kinase activity, protein metabolism, protein modulation, cell structure/cytoskeleton, and transcriptional regulation.

Apoptosis is associated with a number of morphological changes; including cell shrinkage, nuclear condensation, membrane blebbing, caspase activation and DNA fragmentation [25]. Apoptosis has been implicated in both liver damage and cancer development, and HCV-induced liver injury may be mediated by direct cytopathic effects of the virus [4;26;27]. The causes of virus-associated liver injury observed in chronically infected patients are unknown. LB-piVe [28], an HCV genotype 1b plasma isolate that was adapted to grow in VeroE6 cells and induces a strong cytopathic effect in Huh7.5 cells, was used for the analysis of virus-induced apoptosis. Total RNA from LB-piVe-infected Huh7.5 cells was used to screen a human apoptosis PCR array for gene expression profiling. Tumor necrosis factor (TNF), caspase activators, caspases, Fas, and p53, among other differentially regulated genes, were upregulated in virus-infected liver cells. Our findings represent a portrait of genomic changes in HCV-infected cells, and confirm the involvement of previously identified apoptosis pathways in HCV infection and subsequent liver injury. These data reveal new insights into the molecular and immunologic mechanisms governing HCV-mediated apoptosis, induced by genotype 1b, and will be useful for the identification of novel antiviral or antifibrotic targets.

## Materials and Methods

### Cell culture

LB-persistently infected VeroE6 cells (LB-piVe; [28]) were maintained in complete Dulbecco’s modified Eagle’s medium (DMEM; Life Technologies, Grand Island, NY, USA) containing 10% heat-inactivated Fetal Bovine Serum (FBS; Thermo Scientific HyClone, Logan, UT, USA) at 37°C with 5% CO_2_. Huh7.5 cells were provided by C.M. Rice (Rockefeller University, NY) and maintained in complete DMEM containing 10% FBS and non-essential amino acids (Life Technologies, Grand Island, NY, USA). FBS was screened by RT-PCR to ensure the absence of bovine viral diarrhea virus (BVDV).

### Preparation and quantitation of LB-piVe virus

LB-piVe were created by transfecting VeroE6 cells with a plasmid (pVA) encoding adenovirus-associated VA RNAI, as described previously [28]. Briefly, VeroE6 cells were transiently transfected with pVA, a plasmid encoding adenovirus VA RNA_I_, to inhibit interferon pathways. At 24 hr post-transfection, the cells were infected with patient plasma, identified as LB, a genotype 1b strain. Seven days post-infection (dpi), the cells were divided 1:6 and re-transfected with pVA. The passage and transfection process was repeated weekly for 20 weeks, without re-infection [28]. After 20 weeks, the cells were passaged weekly without transfection. Two years later the cells were expressing virus and were passaged weekly for a total of 4 yrs more. Persistently infected cells (LB-piVe) were used to grow virus for infectious stocks. Briefly, flasks (T75) containing LB-piVe cells and culture medium were frozen (at -80°C) and thawed 3 times. The cell debris was removed by centrifugation and subsequent filtering (0.45 μm), and filter-clarified culture supernatants were obtained and used as LB-piVe virus stock. LB-piVe viral titers were measured by titration on naïve Huh7.5 cells, as described [28]. The 50% tissue culture infectious dose (TCID_50_) was calculated using the method of Reed and Muench [29]. UV-inactivated virus was obtained from the same virus stock in conditioned medium. After exposure to UV light (254-nm) for 60 seconds, using a Stratalinker 2400 (Agilent Technologies, Santa Clara, CA), the medium served as the “mock infection”, a non-infectious control in the same conditioned medium. Serial dilutions of the UV-inactivated virus were tested for infectivity in naïve Huh7.5 cells as described [28]. Therefore, the conditioned media contained virus even though it was not infectious. All LB-piVe infections were carried out by infecting naïve Huh7.5 cells at MOI = 0.01 unless indicated.

### Electron microscopy

LB-piVe infected cells were harvested four days post-infection, washed twice with PBS, and fixed by incubation for 48 hours in 4% paraformaldehyde and 1% glutaraldehyde in 0.1 M phosphate buffer pH 7.2. Next, cells were washed in PBS and post-fixed by incubation for 1 hour with 1% osmium tetroxide (Electron Microscopy Biosciences, Hatfield, PA, USA) and dehydrated in a graded series of ethanol solutions. Cell pellets were embedded in Epon resin (Sigma-Aldrich Corp., St. Louis, MO, USA) which was allowed to polymerize for 48 hours at 60°C. Ultrathin sections were cut, stained with 5% uranyl acetate and 5% lead citrate, and deposited on electron microscopy grids coated with collodion membrane, for examination under a Jeol 1230 transmission electron microscope [(TEM); Tokyo, Japan] connected to a Gatan digital camera driven by Digital Micrograph software (Gatan, Pleasanton, CA, USA).

### Apoptosis induction

Apoptotic Huh 7.5 cells were produced by treatment with 100 nM Staurosporine (R&D Systems Inc., Minneapolis, MN, USA) or 3μM Gambogic Acid [30;31] for 16 hours at 37°C. For the immunoblotting experiments, apoptosis was induced with 1μM gambogic acid or 200 ng/ml of actinomycin D for 24 hr.

### Flow cytometry analyses

All flow cytometry studies were performed on a FACS Calibur cytometer (BD Biosciences, San Diego, CA, USA) with data analysis conducted using FlowJo 7.6.3 software (Tree Star, Inc., Ashland, OR, USA).

### Detection of HCV NS5A

Cells were harvested by incubation with Accutase^™^ Cell Detachment Solution (BD Biosciences, San Jose, CA, USA), pelleted (1000 rpm for 5 minutes) and washed twice with ice cold PBS. Fixation and permeabilization was carried out using the BD Cytofix/Cytoperm TM fixation/permeabilization kit (BD Biosciences, San Jose, CA, USA). After an incubation of 30 minutes at 4°C, cells were stained with 0.4 μg (per 10^6^ cells) of mouse anti-HCV NS5A monoclonal antibodies (Austral Biologicals, San Ramon, CA, USA) and Alexa Fluor^®^ 488-conjugated goat anti-mouse IgG (H+L) (Life Technologies, Grand Island, NY, USA) diluted 1/1000.

### Detection of Caspase-3 and Fas

Cells were harvested, fixed and permeabilized as described above. Fas-expressing cells were stained with 0.25 μg (per 10^6^ cells) of PE-Mouse anti-human CD95 (BD Biosciences, San Jose, CA, USA). Caspase-3 expressing cells were stained with 0.25 μg (per 10^6^ cells) of FITC rabbit anti-active caspase-3 antibodies (BD Biosciences, San Jose, CA, USA). For the caspase inhibition assay, cells were treated or mock treated with benzyloxycarbonyl-Val-Ala-Asp-fluoromethylketone (Z-VAD-FMK, BD Biosciences, San Jose, CA, USA), a broad-spectrum caspase inhibitor, at 20 μM for 16 hr at 37°C.

FITC rabbit IgG isotype (eBioscience Inc., San Diego, CA, USA) and Alexa Fluor 488 PE-mouse IgG2b K isotype (eBioscience Inc., San Diego, CA, USA) were used as controls. All flow cytometry studies were performed on a FACS Calibur cytometer (BD Biosciences, San Diego, CA, USA) with data analysis conducted using FlowJo 7.6.3 software (Tree Star, Inc., Ashland, OR, USA).

### Phycoerythrin (PE) Annexin V staining

To determine expression levels of phosphatidylserine at the cell surface, cells were stained with PE Annexin V in a buffer containing 7-Amino-Actinomycin D (7-AAD) following manufacturer’s protocol (PE Annexin V Apoptosis Detection Kit I, BD Biosciences, San Jose, CA, USA). Cells were harvested and washed twice with ice cold PBS. 10^5^ cells were incubated with 5 μl of PE Annexin V and 5μl of 7-AAD for 15 minutes at room temperature.

### PCR Arrays

LB-piVe infected cells were harvested four days post-infection and washed twice with ice cold PBS. Total RNA was purified using the RNeasy Mini Kit (Qiagen, Valencia, CA). The concentration and purity of the extracted RNA was determined using a NanoDrop 1000 Spectrophotometer (Thermo Fisher Scientific Inc., Waltham, MA). One μg of purified RNA was used per PCR Array. Genomic DNA elimination was performed by incubating samples for 5 min at 42°C according to manufacturer’s recommendations (Qiagen, Valencia, CA). Next, samples were placed on ice for >1 minute. The Reverse transcription mix was added and the reaction proceeded for 15 min at 42°C. The reaction was stopped by incubating at 95°C for 5 min. After adding RNase-free water to each reaction, samples were mixed with the RT^2^ SYBR Green master mix and aliquoted into the PCR Array plates (Human Apoptosis RT2 Profiler PCR Array, Qiagen, Valencia, CA, USA). Plates were sealed with optical adhesive films and placed in the real-time cycler (Applied Biosystems 7700, Applied Biosystems Foster City, CA). Cycling conditions were as follows: 10 minutes at 95°C and then 40 cycles consisting of 15 seconds at 95°C and 1 minute at 60°C. The CT values were used to analyze the data. Relative expression of genes was determined using data from the real-time cycler (CT values) and the ΔΔCT method *(RT*^*2*^ *Profiler PCR Array Handbook*, *Sabiociences*, *11/2011)*. Fold changes in gene expression were calculated by comparing gene expression in LB-piVe infected cells to that in UV-inactivated LB-piVe infected cells using the Web-Based PCR Array Data Analysis from Sabiociences (Valencia, CA, USA).

### Immunoblot analysis

Cells were harvested, washed with ice-chilled PBS, and then lysed with RIPA lysis buffer [25 mM Tris-HCl (pH 7.6), 150 mM NaCl, 1% NP-40, 1% sodium deoxycholate, 0.1% SDS] containing protease inhibitors (Complete protease inhibitor cocktail; Roche Applied Science, Indianapolis, IN, USA) on ice. Cell extracts were clarified by centrifugation (15 min at 4°C at 13,000 rpm) and total protein concentration was measured using Bio-Rad Protein Assay (Bio-Rad, Hercules, CA, USA) following manufacturer instructions. Total proteins (50 μg) were resolved in Novex 4–20% Tris-glycine polyacrylamide gels (Life Technologies, Grand Island, NY, USA), under reducing conditions. Subsequently, proteins were transferred to Hybond ECL membranes (GE Healthcare Bio-Sciences Pittsburgh, PA, USA), blocked in 5% non-fat Milk in PBS Buffer, and incubated with 1:1000 dilutions of primary antibodies (Cell Signaling Technologies, Inc., Danvers, MA, USA) against: caspase-3, cleaved human poly (ADP-ribose) polymerase (PARP1), Bcl-2-interacting killer (BIK), Bcl-xL and loading control glyceraldehyde-3-Phosphate Dehydrogenase (GAPDH; Trevigen Inc., Gaithersburg, MD). After washing with PBS-Tween, membranes were probed with horseradish peroxidase-conjugated secondary antibodies (Kirkegaard & Perry Laboratories Inc., Gaithersburg, MD). The SuperSignal West Pico or Femto Chemiluminescence Substrate kits (Life Technologies, Grand Island, NY) were used for detection as recommended by the manufacturer.

### Pathway analysis

Pathway analysis was performed using the Pathway Studio 9 Software (Ariadne Genomics, Rockville, MD). Protein interaction maps were generated and colored according to the level of gene expression (red: up-regulated genes; blue: down-regulated genes; grey: no change).

### Accession numbers

Sequences can be accessed from GenBank through the NCBI website: LB-piVe (FJ976045, FJ976046 and FJ976047).

## Results

### Ultrastructural changes in LB-piVe infected Huh7.5 cells show markers of apoptosis

**Fig 1:**
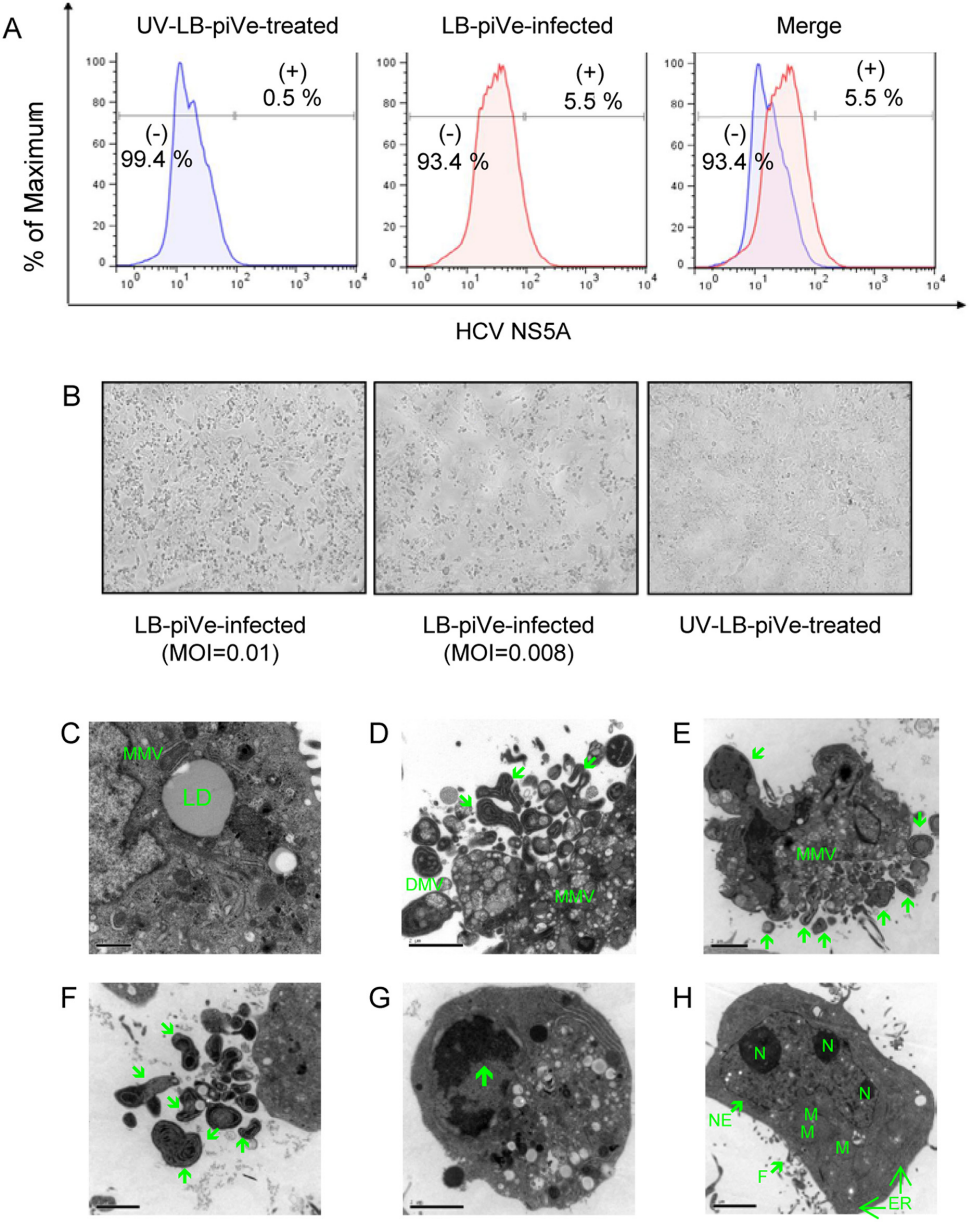
Ultrastructural changes in LB-piVe infected Huh7.5 cells. **(A)** Huh7.5 cells were infected with LB-piVe (center) or with mock infection, UV-LB-piVe (left). Four days post-infection, cells were fixed, permeabilized, stained with anti-HCV NS5A, developed with anti-mouse antibodies conjugated with Alexa Fluor 488, and analyzed by flow cytometry. Numbers indicate the percentage of HCV NS5A-positive and -negative cells. **(B)** Light microscopy of LB-piVe and UV-LB-piVe infected cells demonstrating that CPE was an effect of live virus infection. MOI: multiplicity of infection. **(C-G)** LB-piVe infected or **(H)** UV-LB-piVe treated cells were fixed at 4 days post-infection, embedded in Epon resin and deposited on grids coated with collodion membrane for examination under a Jeol 1230 transmission electron microscope (TEM). Mitochondria (M); Nucleolus (N); Nuclear Envelope (NE); Filopodia (F); Endoplasmic Reticulum (ER); Lipid droplet (LD); Multiple-membrane vesicles (MMV). Green arrows denote markers of apoptosis.

We previously reported that an HCV genotype 1b virus present in the plasma of an infected patient established a persistent infection in VeroE6 cells, demonstrating a mild cytopathic effect (CPE). Persistently infected VeroE6 cells, termed LB-piVe cells, were passaged weekly for two years and maintained in culture for more than 6 years [28]. The virus rescued from LB-piVe cells induced strong CPE when incubated with the HCV-permissive Huh7.5 human hepatoma cell line, while UV-inactivated LB-piVe supernatant did not [28]. To examine the effect of HCV infection at the sub-cellular level, Huh7.5 cells were infected (MOI = 0.01) with LB-piVe or with a mock infection, UV-inactivated LB-piVe (UV-LB-piVe), for 4 days. We verified the presence of viral RNA by RT-PCR (data not shown) and intracellular expression of HCV NS5A by flow cytometry (Fig 1A). We found that approximately 5.50% of the Huh7.5-LB-piVe infected cell population showed an increase in fluorescence intensity (Fig 1A, center panel), compared to only a 0.53% of background staining in UV-inactivated LB-piVe-treated cells (UV-LB-piVe; Fig 1A, left panel).

When visualized by light microscopy, LB-piVe-infected cells (Fig 1B, left and center panels), showed signs of gross cell death that was directly related to the input MOI. No CPE was observed in those cells that were infected with the same volume of the mock-infected, UV-inactivated virus (Fig 1B, right panel), demonstrating that CPE was an effect of live virus infection. Ultrastructural analysis of Huh7.5-LB-piVe infected cells by transmission electron microscopy (TEM) showed virus-induced alterations of cell structures revealed by the presence of a membranous web composed of multiple-membraned vesicles (MMV) and large lipid droplets (LD; Fig 1C). Interestingly, signs of apoptosis, such as membrane blebbing, budding apoptotic bodies (Fig 1D, 1E and 1F, green arrows) and crescent-shaped chromatin aggregation (Fig 1G, green arrow) could also be visualized. No signs of apoptosis were visualized in UV-LB-piVe -infected cells (Fig 1H).

### Membrane expression of phosphatidylserine in LB-piVe infected cells is revealed by positive staining for Annexin V

**Fig 2:**
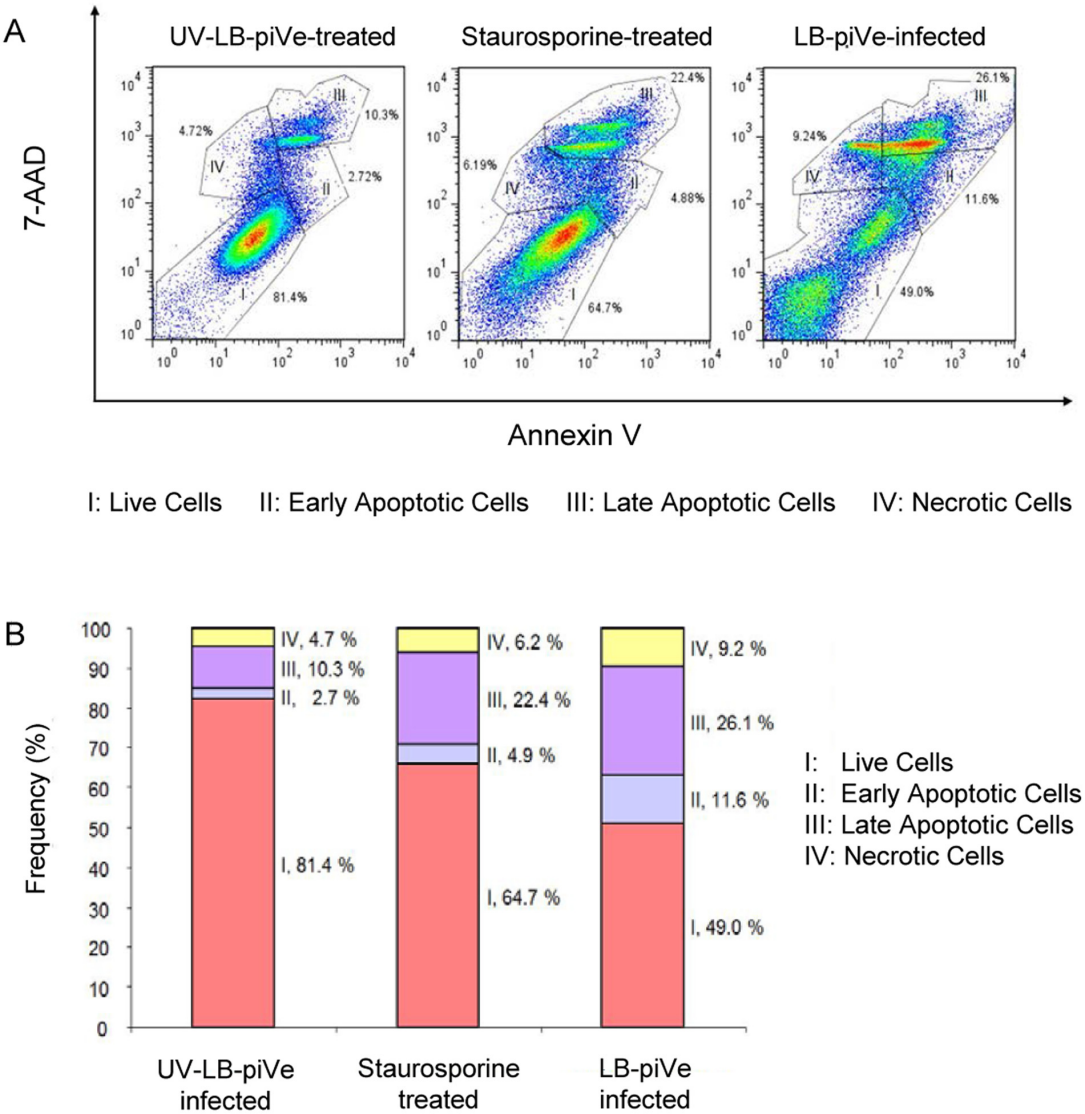
LB-piVe infection triggers exposure of phosphatidylserine on the surface of infected cells. (A) UV-LB-piVe (left panel) or LB-piVe infected (right panel) cells and Staurosporine-treated (center panel) were incubated with PE Annexin V in a buffer containing 7-Amino-Actinomycin D (7-AAD), and analyzed by flow cytometry. Numbers in the quadrants indicate the percentage of cells in the corresponding areas. (B) Representation of the frequency of live, early apoptotic, late apoptotic and necrotic cells from (A).

Changes in the plasma membrane are one of the first characteristics of the apoptotic process detected in living cells. Upon induction of apoptosis, rapid alterations in the organization of phospholipids in most cell types occur, leading to exposure of phosphatidylserine (PS) on the cell surface. PS can be detected through high affinity binding of fluorochrome-labeled Annexin V. The combination of phycoerythrin (PE) Annexin V and 7-Amino-Actinomycin D (7-AAD), which has a high DNA binding constant and is efficiently excluded by intact cells, enables the differentiation between apoptotic and necrotic cells. In this context, viable cells are not stained with Annexin V or 7-AAD; apoptotic cells are positive for Annexin V and negative for 7-AAD, and necrotic cells stain positive for both. To further explore the process of LB-piVe induced apoptosis, virus-infected cells were harvested, and then incubated with PE Annexin V in a buffer containing 7-AAD (Fig 2).

Flow cytometry analysis revealed that UV-LB-piVe-treated cells were primarily negative for PE Annexin V and 7-AAD staining, demonstrating that 81.4% of the cells were viable (I) as indicated in Fig 2A (left panel), and Fig 2B. A minor population of cells were found to be PE Annexin V and 7-AAD positive indicating that they were already dead (IV; necrotic, 4.7%) or early/late apoptotic (II or III; 13%). When cells were treated with the apoptosis-inducer, Staurosporine (Fig 2A, center panel and Fig 2B), only 64.7% were live cells with 27.3% undergoing apoptosis. Interestingly, 49% of the LB-piVe-infected Huh7.5 cells remained alive at 5 dpi (Fig 2A, right panel and Fig 2B), with a large percentage undergoing apoptosis (37.7%). This suggests that virus infection, replication and/or spread are responsible for apoptosis in hepatocytes because the UV-LB-piVe did not induce apoptosis (13%) to the same extent as the infectious virus. The infectious virus induced cell death by necrosis (9.2%; Fig 2A, right panel and Fig 2B) two-fold over the UV-LB-piVe-infected cells (4.7%; Fig 2A, left panel and Fig 2B). This may represent cells that have lysed due to late phase apoptosis or release of cytokines into the supernatant. However, induction of TNF is probably not responsible for necrosis as treatment of LB-piVe-infected Huh7.5 cells with TNF did not induce apoptosis or necrosis (data not shown).

### Gene profiling analysis of LB-piVe infected cells using a human apoptosis PCR array

Chronic HCV infection is associated with progressive liver injury and subsequent development of fibrosis and cirrhosis. The mechanism of liver damage induced by HCV is still under investigation and may comprise apoptosis and immune-mediated processes involving necrosis or autophagy [32–34]. To characterize the mechanism of apoptosis induced by LB-piVe in Huh7.5 cells, we used a human apoptosis PCR array to screen differentially expressed genes implicated in the apoptotic signaling pathways. The array consisted of 84 key genes involved in programmed cell death, 5 housekeeping genes, a genomic DNA control, reverse-transcription controls, and positive PCR controls. Genes that showed a 2-fold or higher change in expression in two independent experiments were considered to be significantly upregulated by viable LB-piVe virus. No genes were found to be significantly down-regulated.

A list of the genes analyzed in this study, grouped according to their biological functions, together with the fold change in expression, is provided in Tables 1 and 2.

**Table 1:** Expression profiles of host genes involved in apoptosis regulation.

Description	Annotation	Gene Symbol	Fold Change
**BIR Domain Proteins**	NLR family, apoptosis inhibitory protein	NAIP/BIRC1	2.339
	Baculoviral IAP repeat containing 6	BIRC6	3.9096
**BCL2 and BAG Domain Proteins**	BCL2-associated athanogene 3	BAG3	2.2737
	B-cell CLL/lymphoma 2	BCL2	-0.384
	BCL2-related protein A1	BCL2A1	3.1208
	BCL2-like 11 (apoptosis facilitator)	BCL2L11	14.9691
	B-cell CLL/lymphoma 10	BCL10	4.3854
	Myeloid cell leukemia sequence 1 (BCL2-related)	MCL1	2.9465
**Death Domain Proteins**	CASP2 and RIPK1 domain containing adaptor with death domain	CRADD	2.2026
	Tumor necrosis factor receptor superfamily, member 1A	TNFRSF1A	2.5923
	Death-associated protein kinase 1	DAPK1	8.588
	TNFRSF1A-associated via death domain	TRADD	3.8411
**TNF/TNFR Domain Proteins**	Tumor necrosis factor	TNF	6.252
	Fas (TNF receptor superfamily, member 6)	FAS/CD95	2.0093
	Lymphotoxin alpha (TNF superfamily, member 1)	LTA	6.3595
	Tumor necrosis factor receptor superfamily, member 9	TNFRSF9	-0.4293
	Tumor necrosis factor (ligand) superfamily, member 10	TNFSF10	2.0727
	Tumor necrosis factor receptor superfamily, member 10a	TNFRSF10A	0.6248
	Tumor necrosis factor receptor superfamily, member 10b	TNFRSF10B	1.0503
**DNA Damage**	C-abl oncogene 1, non-receptor tyrosine kinase	ABL1	2.8095
	Cell death-inducing DFFA-like effector a	CIDEA	2.5333
	Tumor protein p53	TP53	3.6833
	Tumor protein p73	TP73	7.3057
	Tumor protein p53 binding protein, 2	TP53BP2	2.8827
	DNA fragmentation factor, 45kDa, alpha polypeptide	DFFA	2.4
**Other Genes**	Nucleolar protein 3 (apoptosis repressor with CARD domain)	NOL3	2.5342
	Bifunctional apoptosis regulator	BFAR	3.0149
	BCL2/adenovirus E1B 19kDa interacting protein 1	BNIP1	2.0093
	BCL2/adenovirus E1B 19kDa interacting protein 3	BNIP3	2.1125
	BCL2/adenovirus E1B 19kDa interacting protein 3-like	BNIP3L	2.6475
	V-raf murine sarcoma viral oncogene homolog B1	BRAF	3.4446
	Insulin-like growth factor 1 receptor	IGF1R	6.1758
	V-akt murine thymoma viral oncogene homolog 1	AKT1	2.4582
	BCL2-associated agonist of cell death	BAD	4.1191
	BCL2-antagonist/killer 1	BAK1	2.356
	BH3 interacting domain death agonist	BID	2.0824
	BCL2-interacting killer (apoptosis-inducing)	BIK	44.5489

**Table 2:** Expression profiles of Caspase and Caspase Regulator Genes.

Description	Annotation	Gene Symbol	Fold Change
**Caspases**	Caspase 1, apoptosis-related cysteine peptidase	CASP1	0.6235
	Caspase 2, apoptosis-related cysteine peptidase	CASP2	1.2663
	Caspase 3, apoptosis-related cysteine peptidase	CASP3	5.3689
	Caspase 4, apoptosis-related cysteine peptidase	CASP3	1.9693
	Caspase 5, apoptosis-related cysteine peptidase	CASP5	0.9462
	Caspase 6, apoptosis-related cysteine peptidase	CASP6	4.3696
	Caspase 7, apoptosis-related cysteine peptidase	CASP7	0.9813
	Caspase 8, apoptosis-related cysteine peptidase	CASP8	2.8185
	Caspase 9, apoptosis-related cysteine peptidase	CASP9	3.6762
	Caspase 10, apoptosis-related cysteine peptidase	CASP10	1.2684
	Caspase 14, apoptosis-related cysteine peptidase	CASP14	0.8269
	CASP8 and FADD-like apoptosis regulator	CFLAR	2.2841
	PYD and CARD domain containing	PYCARD	2.9924
**Caspase Activators**	Apoptotic peptidase activating factor 1	APAF1	4.7973
	BCL2-associated X protein	BAX	1.1592
	BCL2-like 10 (apoptosis facilitator)	BCL2L10	2.0208
	Caspase recruitment domain family, member 6	CARD6	2.2971
	Caspase recruitment domain family, member 8	CARD8	4.481
	Nucleotide-binding oligomerization domain containing 1	NOD1	4.8186
**Caspase Inhibitors**	CD27 molecule	CD27	-0.2632

LB-piVe-infected cells demonstrated significant changes in the expression of several important host genes that are involved in both the extrinsic apoptosis pathway activated through the death receptors, and the intrinsic apoptosis pathway that is dependent on mitochondrial dysfunction [25]. Among the genes with the greatest induction were pro-apoptotic proteins such as BCL2-interacting killer (BIK; 44.5 fold), BCL2-like 11 (BCL2L11; 14.9 fold), Death-associated protein kinase 1 (DAPK1; 8.6 fold), Tumor protein p73 (TP73; 7.3 fold), Lymphotoxin alpha (LTA; 6.3 fold), Tumor necrosis factor (TNF; 6.25 fold), BCL2-associated agonist of cell death (BAD; 4.1 fold), TNFRSF1A-associated via death domain (TRADD; 3.8 fold), and TNF receptor superfamily member 6 (Fas, CD95) (Table 1).

A number of caspases and caspase activators were found to be upregulated in virus-infected cells (Table 2). Caspases 3, 6, 8 and 9, were up-regulated in infected cells (5.4-, 4.4-, 2.8- and 3.7-fold respectively; Table 2). Several caspase activator genes including apoptotic peptidase activating factor 1 (APAF1; 4.8-fold), caspase recruitment domain family, members 6 and 8 (CARD6; 2.3-fold, and CARD8; 4.5-fold), and nucleotide-binding oligomerization domain containing 1 (NOD1; 4.8-fold) were also induced upon infection. In contrast, no differential expression of caspases 2, 4, 5, 7, 10 and 14 was detected (Table 2).

### Functional analysis of regulated genes in LB-piVe infected cells

**Fig 3:**
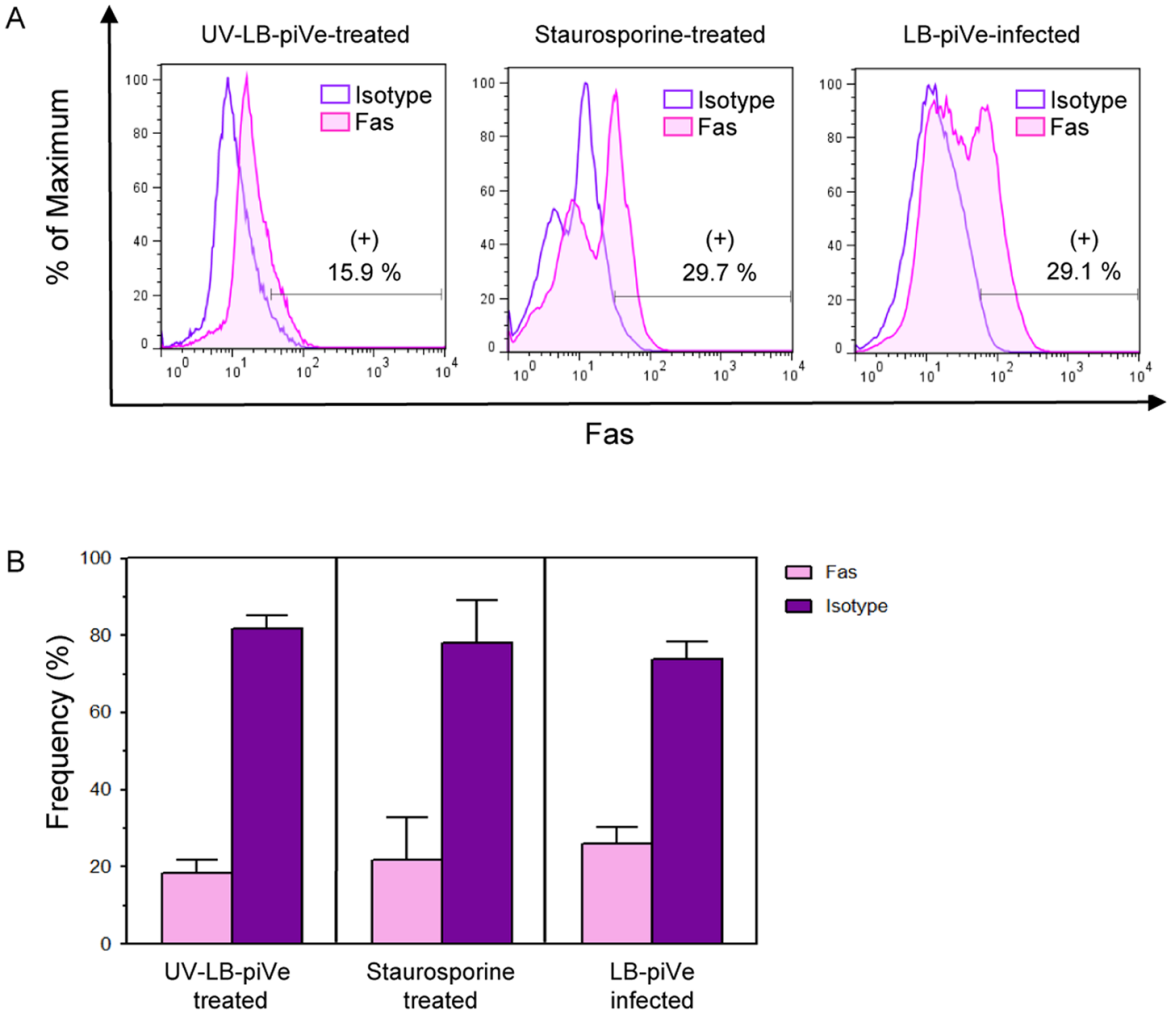
LB-piVe infection up-regulates Fas expression. **(A)** Huh7.5 cells were treated with UV-LB-piVe (left panel), infected with LB-piVe (right panel) or treated with Staurosporine as a positive control for apoptosis (center panel). Four days post-infection, cells were fixed, permeabilized, and then stained with a PE-Mouse anti-human CD95 antibody or a PE Mouse IgG1 Kappa Isotype Control. Stained cells were analyzed using a FACS Calibur cytometer. Data were processed with the FlowJo 7.6.3 software. Numbers indicate the percentage of Fas-positive and Fas-negative cells. **(B)** Representation of the frequency of Fas-positive and Fas-negative cells from **(A).** Frequency values are means ± SD, representative of 2 different experiments.

To confirm that the differentially expressed genes were also up-regulated at the protein level, we randomly selected some of those genes to explore their expression using flow cytometry analysis. Since the PCR array showed that Fas receptor (CD95) was up-regulated twofold in virus–infected cells (Table 1), we stained both LB-piVe-infected and UV-LB-piVe-treated Huh7.5 cells with a PE-mouse anti-human Fas antibody. We observed that 29.1% of the virus-infected cells expressed Fas as shown in Fig 3A (right panel), and Fig 3B. The level of Fas expression was comparable to our positive control for apoptosis, the Staurosporine–treated cells (29.7%; Fig 3A, center panel and Fig 3B). In contrast, only 15.9% of the UV-LB-piVe-treated cells stained positive for Fas (Fig 3, left panel and Fig 3B), which is consistent with the PCR array (Table 1).

**Fig 4:**
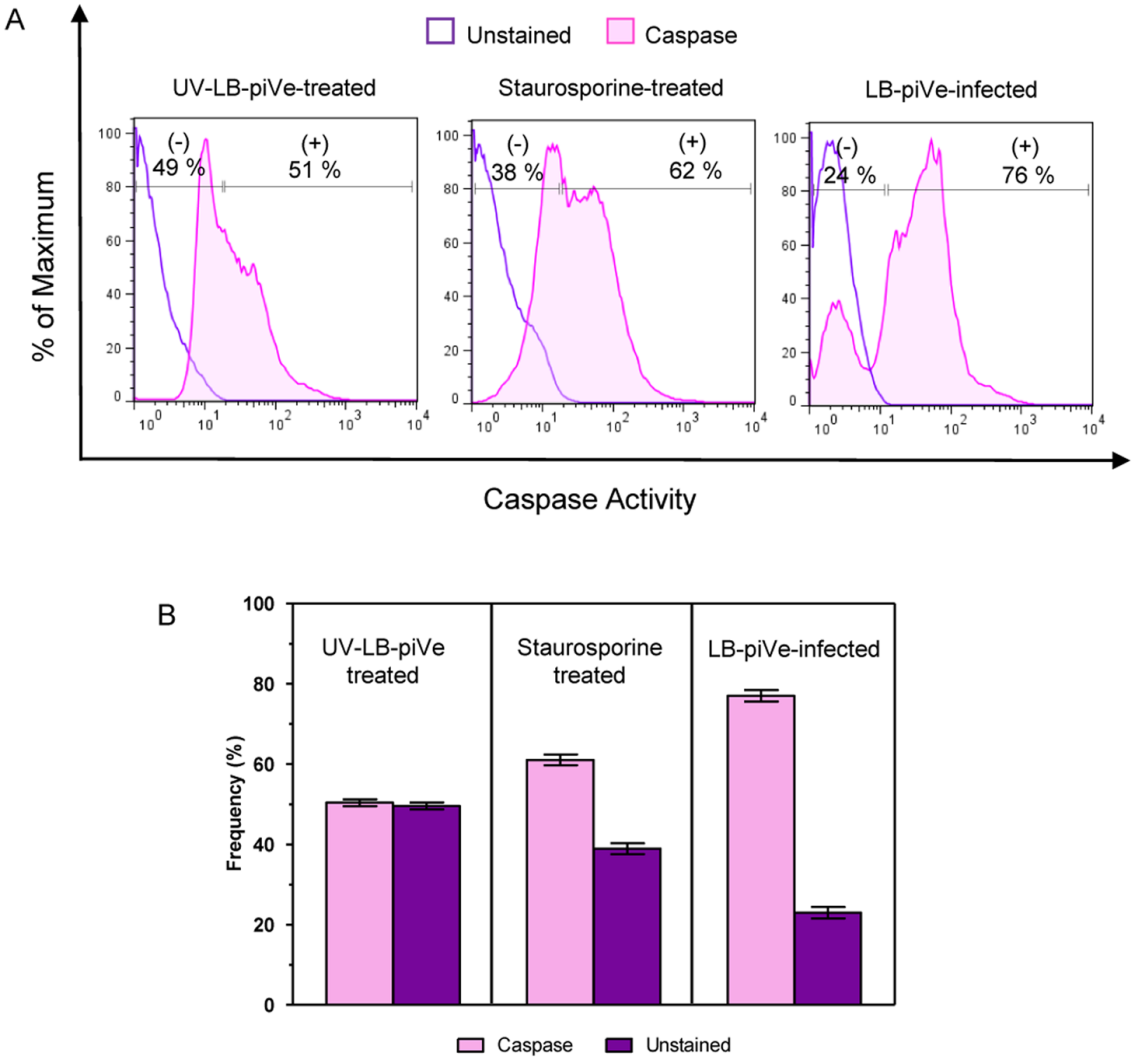
LB-piVe infection is associated with caspase activation. **(A)** Intracellular caspase detection was performed by staining live Huh7.5 cells with FITC-conjugated ApoStat and analyzing by flow cytometry. Cells were evaluated at 4 days post-infection or treatment. UV-LB-piVe-treated and Staurosporine-treated cells were used as negative and positive controls, respectively. Data were processed with the FlowJo 7.6.3 software. Numbers indicate the percentage of caspase positive and negative cells. **(B)** Representation of the frequency of caspase positive and negative cells from **(A)**. Frequency values are means ± SD, representative of 2 different experiments.

The Fas pathway can potentially induce apoptosis by activation of caspase-8 and caspase-3. Because Fas expression was up-regulated in LB-piVe-infected cells (Table 1 and Fig 3), it was of interest to determine if caspase proteins were similarly activated in these cells. Total intracellular caspase activation was examined using a cell-permeable, FITC-conjugated pan-caspase inhibitor (ApoStat, R@D Systems, Inc., Minneapolis, MN, USA). Huh7.5 cells were infected with either LB-piVe or treated with UV-LB-piVe, or Staurosporine. Cells were then harvested, washed and stained with ApoStat. It should be noted that ApoStat only binds to active caspases and the unbound ApoStat is washed away. When assayed by flow cytometry, 76% of the LB-piVe-infected cells expressed active caspases (Fig 4A, right panel, and Fig 4B) compared to 51% of the UV-LB-piVe cells (Fig 4A, left panel). Active caspases were stained in 62% of the staurosporine-treated cells (Fig 4A, center panel and Fig 4B).

**Fig 5:**
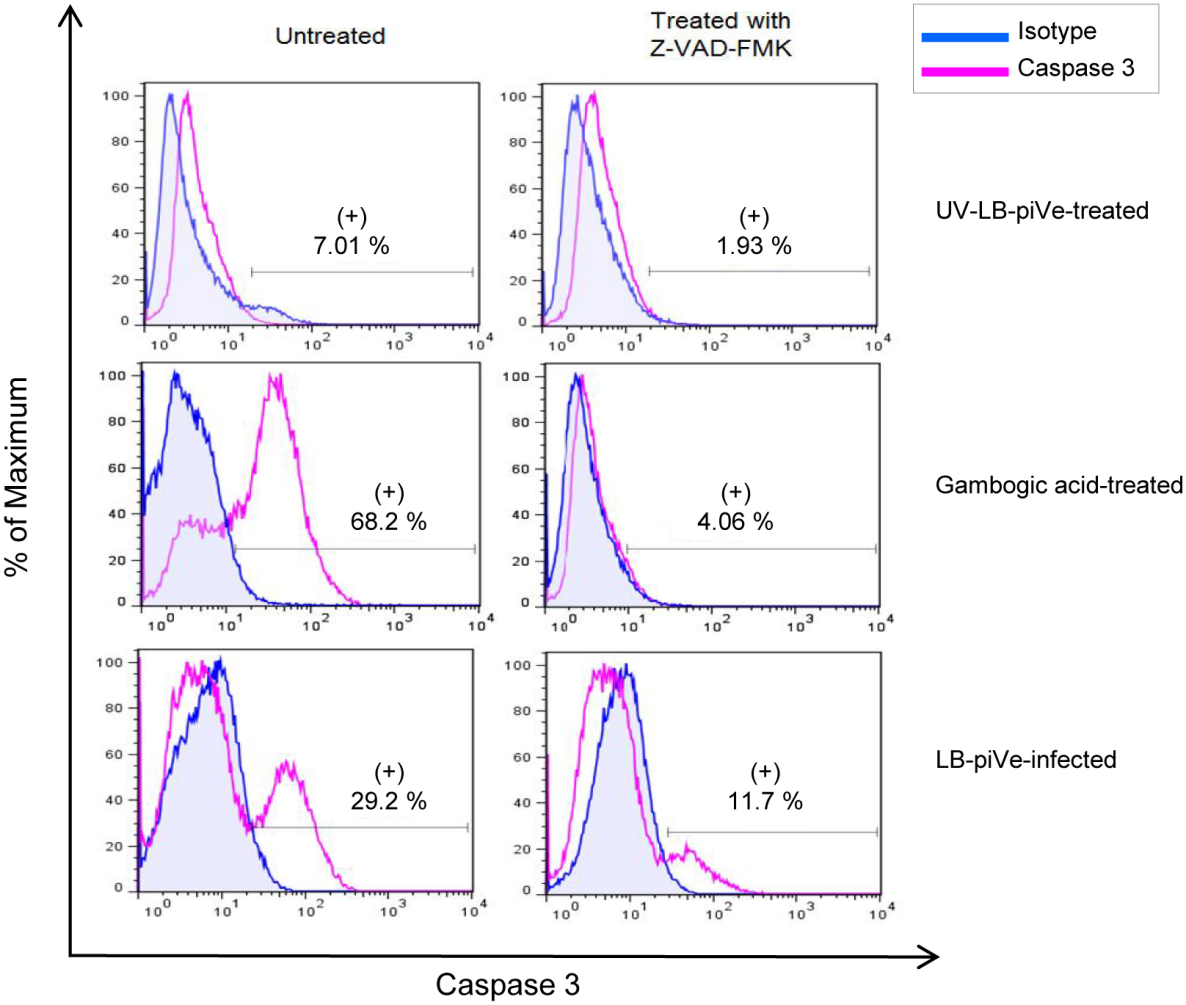
LB-piVe infection up-regulates active caspase-3 expression. **(A)** Huh7.5 cells were UV-LB-piVe-infected (left panel), LB-piVe infected (right panel) or treated with Staurosporine (center panel). At day 3 post-infection, cells were treated (right panel) with Z-VAD-FMK at 20 μM for 16 hr at 37°C. The following day, cells were fixed/ permeabilized, stained with FITC Rabbit Anti-Active Caspase-3 antibodies or FITC mouse isotype control and analyzed by flow cytometry. Data were processed with the FloJo 7.6.3 software. Numbers indicate the percentage of caspase-3 positive and negative cells. **(B)** Representation of the frequency of caspase-3 positive and negative cells from **(A).**

Further characterization of caspase 3 activity was observed in HCV-infected cells, using an inhibitor of caspase 3 activity (Z-VAD-FMK) to show specificity. Cell staining with a rabbit anti-active caspase-3 antibody showed that caspase-3 activity was four times higher in LB-piVe-infected cells (29.2%; Fi5, left bottom panel) than in the UV- LB-piVe-treated cells (7.01%; Fig 5, left top panel). In this experiment, gambogic acid was used to induce apoptosis and activate caspase 3 for use as a positive control. Gambogic acid-treatment yielded 68.2% of cells expressing activated caspase-3 (Fig 5, left center panel). The addition of the broad caspase inhibitor Z-VAD-FMK caused a decrease in the amount of active caspase 3 in all cases (Fig 5, right panels), as expected. Some residual caspase 3 activity remained in both Gambogic acid-treated cells (4%, Center, right) and LB-piVe-treated cells (11.7%), suggesting that caspase 3 was activated rapidly after infection and before Z-VAD-FMK treatment.

**Fig 6:**
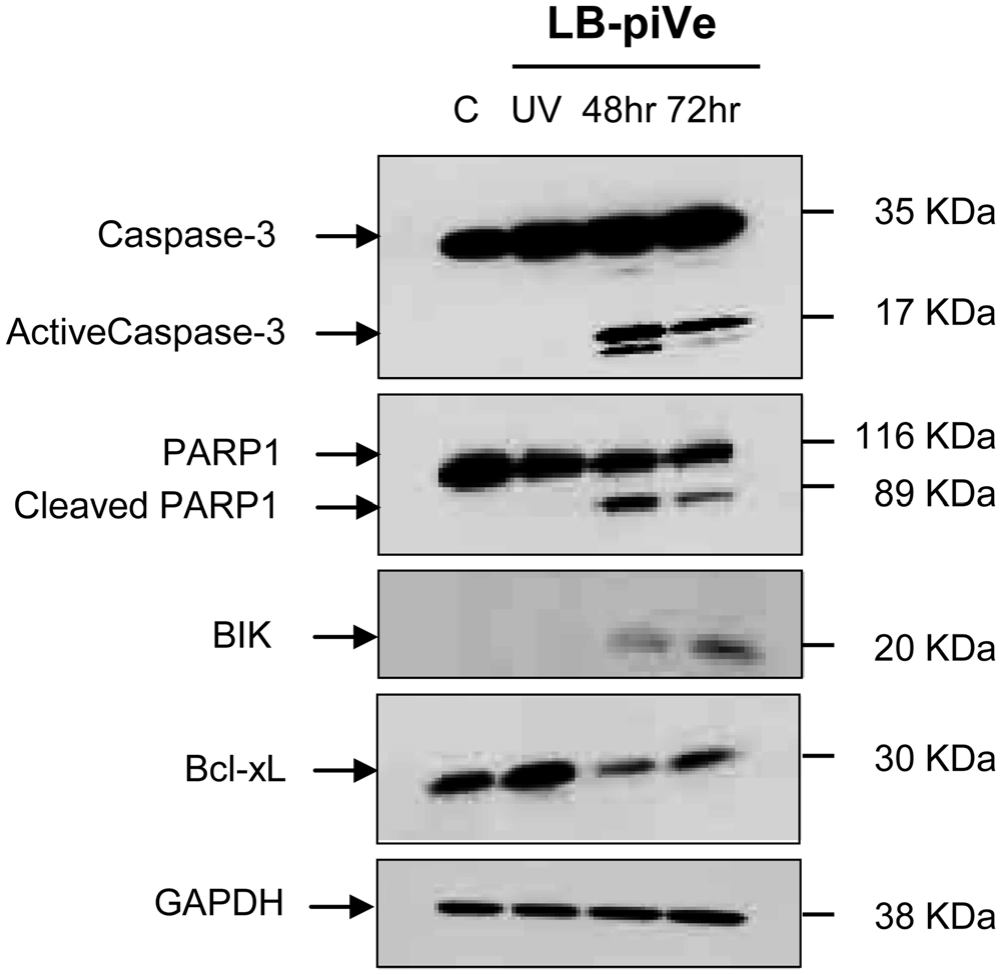
Effect of LB-piVe infection on apoptosis-related genes expression. 50μg of total protein samples were resolved by SDS-PAGE in Novex 4–20% Tris-glycine polyacrylamide gels and then transferred to Hybond ECL membranes. Blots were probed with anti-caspase-3, anti-cleaved PARP1, anti-BIK, and anti- Bcl-xL. Horse radish peroxidase-conjugated secondary antibodies were used for detection and developed with the Femto Chemiluminescence Substrate kit. GAPDH was used as internal loading control. Negative controls: naïve Huh7.5 cells (C), and Huh7.5 cells treated with UV-LB-piVe (UV).

LB-piVe-induced caspase 3 activation was confirmed in lysates of infected cells by immunoblot analysis (Fig 6). The results showed that caspase-3 was processed to generate an active 17-KDa fragment in LB-piVe-infected cells (Fig 6; 48 and 72 hours post-infection) but not in naïve Huh7.5 (C; Fig 6) or UV-LB-piVe-treated cells.

Active caspases cleave human poly (ADP-ribose) polymerase (PARP1) to generate an 89-KDa fragment [35;36]. Caspase 3 was present in Huh7.5 cells, but the activated form of caspase 3 was detected only in cells that were infected with live virus. Therefore, we investigated whether cleaved PARP1 also was present in LB-piVe-infected cells. The 89-KDa fragment was detected by immunoblot in infected cells both at 48 and 72 hours post-infection, but not in naïve Huh7.5 or UV-LB-piVe-treated cells (Fig 6).

Fas and TNF lead to caspase activation in the extrinsic pathway. To expand our analysis of the intrinsic apoptosis pathway [25], we studied expression of two additional proteins in HCV-infected cells: Bcl-2-interacting killer (BIK, 20 KDa) and Bcl-xL (30 KDa). BIK was only detected in cell lysates of LB-piVe-infected cells but not in naïve Huh7.5 or UV-LB-piVe-treated cells (Fig 6). Down-regulation of the anti-apoptotic Bcl-xL, an isoform of BSL2, was observed in LB-piVe-infected cells, consistent with the PCR array observations (Table 1).

### Protein interactions and pathway analysis

**Fig 7:**
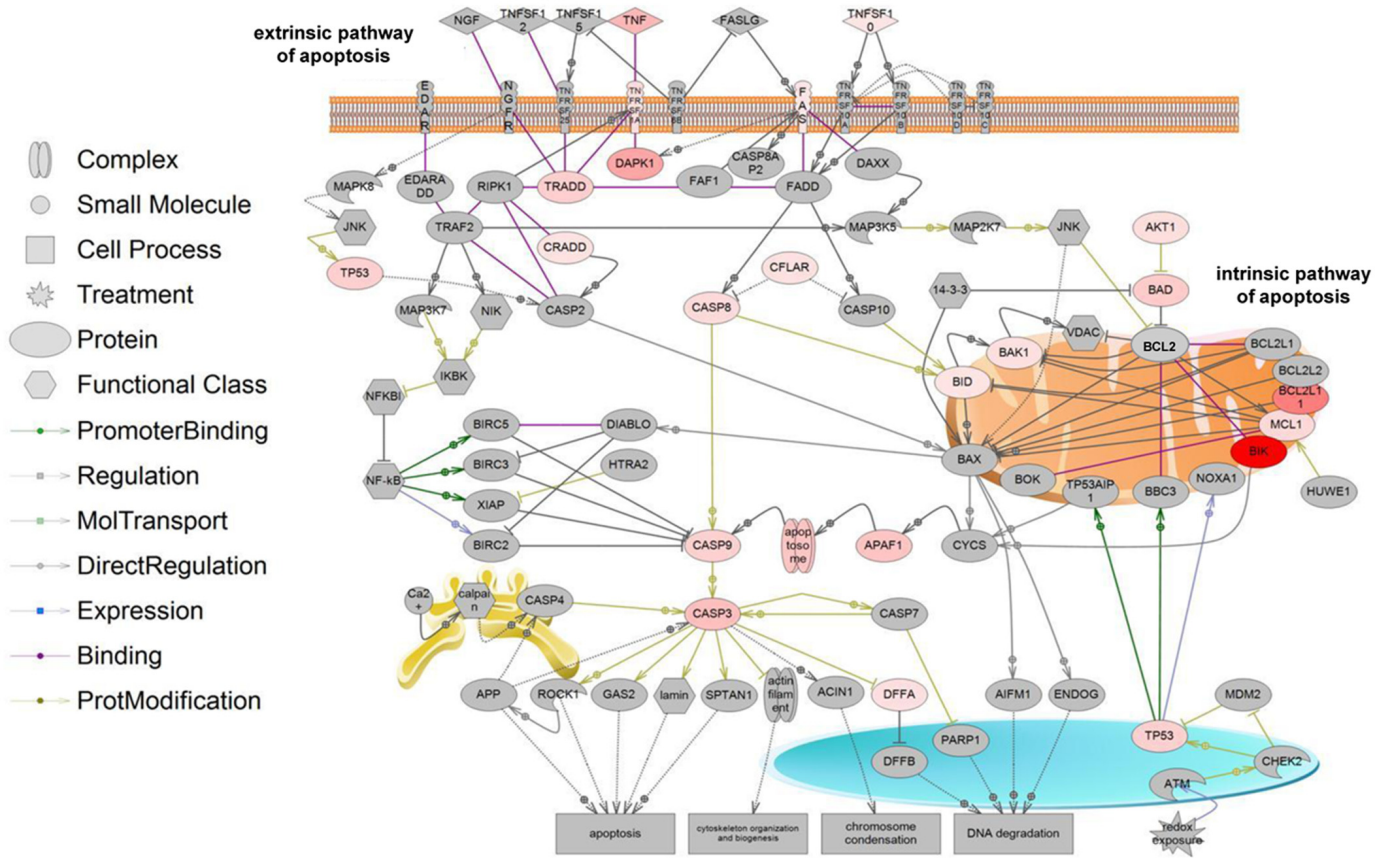
Apoptotic signaling pathways triggered by LB-piVe infection. Pathway analysis was performed with the Pathway Studio 9 software. Using the PCR Array data, a network was generated in which the genes and gene products are represented by nodes. The shapes of the nodes represent the protein’s primary function and the connection lines indicate the type of interaction (colored lines: direct regulation; dotted gray lines: indirect regulation). Color intensity reflects the level of gene expression in LB-piVe infected cells versus control if the fold-change ≥1.2 (red: up-regulated; blue: down-regulated; grey: no change in expression).

To further understand the interactions among the multiple genes regulated during LB-piVe infection of Huh7.5 cells, we analyzed the protein network generated by the PCR array datasets using the Pathway Studio software. Potential biological interactions, cross-talk, and modifications among these differentially expressed proteins are shown in Fig 7. The caspase cascade seems to play an important role in the process, by linking the death receptor (extrinsic) and mitochondrial (intrinsic)-mediated pathways [37–39]. The observation of TNF and Fas up-regulation, two key players in the initiation of apoptosis, together with up-regulation of other genes including, BAD and BIK, located at the mitochondrial membrane, clearly shows that LB-piVe infection triggers an extremely complex process.

The pathway analysis taken with protein expression studies (Figs 2–6) demonstrate that LB-piVe infection is associated with cell apoptosis that may be triggered through both the extrinsic and intrinsic pathways. Furthermore, these data suggest that LB-piVe infection, which results in cytopathic effect, is marked by the induction of apoptosis in Huh7.5 cells. Our data suggests that apoptosis in HCV-infected cells occurs by activation of the extrinsic pathway via Fas-ligand binding and subsequent activation of caspase-3 through a caspase cascade that leads to the cleavage of PARP1, which is known to cause programmed cell death [35;36].

## Discussion

In the last decade, a shift toward therapies that inhibit viral or host proteins essential for HCV replication has become a major focus in drug development research [40;41]. A better understanding of the HCV life cycle and the host processes that trigger HCV-associated pathology is imperative to ensure the development of successful antiviral therapies. Host immune mechanisms and direct effects of HCV proteins have been implicated in hepatitis C viral pathogenesis and apoptosis has been linked to the progression of HCV-associated liver injury [23;26;42–44]. However, the molecular processes and specific cellular factors that determine viral persistence and pathogenesis remain to be elucidated.

Liver fibrosis due to viral infection is one of the leading reasons for liver dysfunction, including liver failure associated with HCV infection. Assessment of liver damage is performed by liver biopsy; the most common method for detection and evaluation of fibrosis. Liver biopsy is invasive, painful and associated with serious complications. Apoptosis of hepatic stellate cells is associated with the regression of fibrosis. Therefore, it is important to delineate the mechanisms involved in cell death and evaluate biomarkers that lead to the progression of viral-induced fibrosis.

In this study, we investigated the changes in human hepatoma gene expression patterns induced by LB-piVe [28], an HCV plasma isolate that adapted to grow in VeroE6 cells, wherein it induced a mild cytopathic effect. To evaluate the strong cytopathic effect of LB-piVe and correlate its eruption with apoptosis, we studied the alterations in cell structures induced by the virus. Huh7.5 cells were infected with LB-piVe virus. We observed that approximately 5.50% of the Huh7.5-LB-piVe infected cells expressed NS5A (Fig 1A, center panel). Interestingly, many LB-piVe-infected cells (Fig 1B, left and center panels), showed signs of gross cell death when visualized by light microscopy. We suspect that we could only detect 5.50% of NS5A-positive cells because cells undergoing apoptosis could not be stained due to massive cell destruction.

Ultrastructural analysis of Huh7.5-LB-piVe infected cells by transmission electron microscopy (TEM) elaborated the presence of a membranous web composed of multi-membranes vesicles (MMV) and large lipid droplets (Fig 1C, 1D and 1E). Our findings are consistent with previous reports which have shown that HCV induces changes in the host-cell membranes, resulting in a membranous web after activation of the autophagy pathway [45–47]. In addition, HCV infection of Huh7 cells induces autophagocytic vacuoles [48;49] and the autophagy machinery is required for HCV replication [50]. Accumulation of autophagosomes is triggered via activation of the unfolded protein response pathway and concomitant up-regulation of the autophagosome marker, LC3 [48]. It has been proposed by Chu et al. that autophagosomes may provide a hidden reservoir for HCV replication; protecting the virus from host innate immune responses [51]. While the autophagy pathways are out of the scope of this study, based on previous studies, we presume that both autophagic and apoptotic pathways are activated and involved in cross-talk, induced by HCV.

However, membrane blebbing, budding apoptotic bodies (Fig 1D, 1E and 1F, green arrows), crescent-shaped chromatin aggregation (Fig 1G, green arrow) could also be observed. These ultrastructural observations, as well as LB-piVe induced changes in the plasma membrane of infected cells revealed that LB-piVe-infected cells were undergoing cell death, suggesting a direct link between virus infection and apoptosis (Fig 2A). Gene expression analysis of infected cells is a powerful tool that can be used to characterize host-cell antiviral responses, including those that may contribute to liver pathology. Previously, the impact of HCV replication on host cell functions has been studied using microarray expression profiling in cells that were transfected with RNA encoding either individual HCV genes, HCV subgenomic or full-length replicons, and in cells infected with the HCV J6/JFH-1 chimera, JC1 [16;18–24]. These studies demonstrated that HCV regulates host genes involved in oxidative stress, apoptosis, lipid metabolism, immunity, proliferation, and intracellular transport [24]. A recent study using an apoptosis-specific PCR array [52] identified BCL2-interacting killer (BIK) mRNA expression as up-regulated, consistent with our results, reported here (Table 1). They showed that expression was also upregulated at the protein level and revealed its role in HCV replication through interaction with NS5B.

Gene expression profiles in liver biopsy tissue from patients with fibrosis and cirrhosis resulting from HCV genotype 3a infection identified significant changes in the expression of genes involved in cell signalling, kinase activity, protein metabolism, protein modulation, cell structure/cytoskeleton, and transcriptional regulation [18]. Most notably, up-regulation of caspase-9 and epithelial membrane protein 1 (EMP1) was associated with fibrosis and Bcl-2-related proline-rich protein (BCL2L12) and programmed cell death protein 1 precursor (PDCD1) was associated with cirrhosis. Here, we report results of the first expression profile screening of hepatocytes infected with HCV genotype 1b using a human apoptosis PCR array containing 84 key genes involved in programmed cell death. Both anti-apoptotic and pro-apoptotic genes; BCL2-interacting killer (BIK; 44.5 fold), Tumor necrosis factor (TNF; 6.25 fold), TNF receptor superfamily member 6 (Fas; 2 fold), BCL2-like 11 apoptosis facilitator (BCL2L11; 14.96 fold), caspase-3 (CASP3; 5.36 fold), caspase-6 (CASP6; 4.36 fold) and caspase-9 (CASP9; 3.67) were among the genes that were upregulated (Tables 1 and 2).

To verify the PCR array results at the protein level, we measured their expression in functional assays. Of special interest was the Fas pathway since it is known to trigger cell apoptosis by activating caspase-9 and then caspase-3 more downstream. Up-regulation of Fas and caspase-3 activities in LB-piVe-infected cells was confirmed by flow cytometry (Figs 3 and 5), consistent with previous findings [23;53].

After activation, caspases can cleave human poly (ADP-ribose) polymerase (PARP1) to generate an 89-kDa fragment [35;36]. Immunoblot analysis revealed the presence of cleaved PARP1 which has a role in DNA repair and maintenance of genome integrity. The presence of cleaved PARP1 is indicative of DNA damage, a key feature of apoptosis. Significant down-regulation of the anti-apoptotic protein Bcl-xL was observed in LB-piVe infected but not in UV-LB-piVe treated cells (Fig 6), suggesting a role for HCV in regulating programed cell death, and consequently determining the fate of infected hepatocytes. The protein network generated during this study (using the PCR array data) clearly shows the association of the two key players in the initiation of apoptosis; TNF and Fas with the caspase cascade and with genes such as BAD and BIK, located at the mitochondrial membrane (Fig 7).

Collectively, the PCR array, the gene expression and functional analyses described here, strongly indicate that LB-piVe infection is associated with programmed cell death. More importantly, the data suggest that both the extrinsic and intrinsic apoptotic pathways are involved in HCV-1b-mediated apoptosis. Further studies will be needed to define all the factors involved in this complex process and to better understand the mechanism of apoptosis in hepatocytes and their role in the progression of fibrosis. The genes identified in this study will provide the basis for new insights into HCV pathogenesis and may possibly help to identify novel therapeutic targets.
